# Association of pan-immune inflammation value, platelet-to-neutrophil ratio and fibrinogen-to-albumin ratio with lower extremity artery disease in patients with type 2 diabetes mellitus: a cross-sectional study

**DOI:** 10.3389/fendo.2025.1739090

**Published:** 2026-01-09

**Authors:** Sheng Zeng, Bin Ou, Zhiyuan Liu, Feng Shi

**Affiliations:** 1 Department of Cardiac and Vascular Surgery, Meizhou People’s Hospital, Meizhou, China; 2 Department of Endocrinology, Meizhou People’s Hospital, Meizhou, China

**Keywords:** fibrinogen-to-albumin ratio, lower extremity artery disease, pan-immune inflammation value, Platelet-to-neutrophil ratio, type 2 diabetes mellitus

## Abstract

**Background:**

Inflammation and coagulation function are considered to be related to atherosclerotic diseases. However, the relationship between comprehensive indices reflecting inflammatory and procoagulant statuses(pan-immune inflammation value (PIV), platelet-to-neutrophil ratio (PNR), and fibrinogen-to-albumin ratio (FAR)) and lower extremity artery disease(LEAD) in patients with type 2 diabetes mellitus (T2DM) is unclear. This research is precisely aimed at studying this issue.

**Methods:**

A total of 9043 patients with T2DM were retrospectively analyzed. PIV, PNR, and FAR were calculated based on monocyte count, neutrophil count, platelet count, lymphocyte count, fibrinogen, and albumin. The relationship between PIV, PNR, and FAR and LEAD was analyzed.

**Results:**

There were 2573 (28.5%) T2DM patients with LEAD and 6470 (71.5%) cases without. The optimal threshold of PIV, PNR, and FAR was 515.86, 43.145, and 0.105 by ROC analysis. There were statistically significant differences in proportions of high PIV, low PNR, and high FAR between T2DM patients with and without LEAD. Logistic regression analysis showed that cigarette smoking(odds ratio(OR): 1.802, 95% confidence interval(CI): 1.506-2.156, *p* < 0.001), hypertension (OR: 1.633, 95% CI: 1.460-1.826, *p* < 0.001), history of cerebrovascular disease (OR: 3.034, 95% CI: 2.678-3.437, *p* < 0.001), and diabetic peripheral neuropathy (OR: 18.983, 95% CI: 15.819-22.780, *p* < 0.001), high PIV (OR: 1.338, 95% CI: 1.181-1.515, *p* < 0.001), low PNR (OR: 2.234, 95% CI: 1.985-2.515, *p* < 0.001), and high FAR (OR: 1.676, 95% CI: 1.493-1.881, *p* < 0.001) were significantly associated with LEAD in T2DM patients.

**Conclusions:**

PIV, PNR, and FAR can serve as potential inflammation- and coagulation-related biomarkers for assessing the risk of LEAD in T2DM patients, thereby providing a reference basis for clinical early screening, risk stratification, and intervention.

## 1 Introduction

Type 2 diabetes mellitus (T2DM) is a common chronic metabolic disease, mainly caused by the combined effect of insulin resistance and insufficient insulin secretion, resulting in elevated blood sugar levels in the body (1). The 2021 Global Burden of Disease Study data shows that the global prevalence of T2DM exceeds 6% (6,059 per 100,000), and it is predicted that by 2030, the global prevalence of T2DM will reach 7,079 per 100,000 (2). Lower extremity artery disease (LEAD) is a common and serious microvascular atherosclerotic disease of T2DM, which can lead to lower extremity ischemia, ulcers and even amputation, significantly increasing the disability rate and mortality rate of patients (3). In patients with T2DM, LEAD refers to a pathological state in which, under the influence of a hyperglycemic environment, atherosclerosis occurs in the lower extremity arteries, the vessel walls thicken, and the lumen becomes narrowed or occluded, thereby causing insufficient blood supply to the lower extremities (4, 5). The clinical symptoms of LEAD are diverse, such as coldness, numbness, and intermittent claudication in the lower limbs in the early stage (6), and pain occurs at rest and may even develop into foot ulcers and gangrene with the condition progresses.

Epidemiological studies have shown that the incidence of LEAD in patients with T2DM is significantly higher than that in non-diabetic populations. The prevalence of LEAD in patients with T2DM exceeds 15% worldwide, and it increases with age and the prolongation of diabetes course (4, 7). Several studies have shown that the incidence of LEAD among T2DM patients is as high as 20%-40% in China (8, 9). In addition, T2DM patients with combined risk factors such as hypertension, hyperlipidemia, and smoking have a higher risk of LEAD (10). The high incidence rate makes LEAD the main cause of lower extremity dysfunction and disability in patients with T2DM, imposing a heavy economic burden on patients’ families and society.

The pathogenic mechanism of LEAD in patients with T2DM is complex, involving multiple factors such as metabolic disorders, inflammatory responses (11), oxidative stress and vascular endothelial function impairment (12), and genetic factors (13). Long-term hyperglycemic conditions can lead to intracellular metabolic disorders and promote the generation of advanced glycation end products (AGEs) by activating the polyol pathway, protein kinase C (PKC) pathway and hexosaccharide pathway (14, 15). AGEs bind to receptors on the cell membrane, triggering inflammatory responses and oxidative stress, damaging vascular endothelial cells, and disrupting the structure and function of the vascular wall (16, 17). At the same time, hyperglycemia can also affect lipid metabolism, leading to oxidative modification of low-density lipoprotein cholesterol (LDL-C), promoting the migration of monocytes and macrophages to the intima of blood vessels, forming lipid streaks, and further developing into atherosclerotic plaques (18, 19). The inflammatory response also plays a key role in the occurrence and development of LEAD. The elevated levels of inflammatory factors such as tumor necrosis factor -α (TNF-α) and interleukin-6 (IL-6) in patients with T2DM can activate vascular endothelial cells, promote the expression of adhesion molecules, mediate the adhesion and migration of white blood cells, and accelerate the process of atherosclerosis (20). The large amount of reactive oxygen species (ROS) produced by oxidative stress can directly damage vascular endothelial cells, inhibit the activity of endothelial nitric oxide synthase (eNOS), reduce the production of nitric oxide (NO), lead to vascular dilation dysfunction, and at the same time promote the oxidation of LDL-C and platelet aggregation, further aggravating vascular lesions (21). In addition, the abnormal proliferation and migration of vascular smooth muscle cells, as well as the remodeling of the extracellular matrix of the vascular wall, are also important pathological bases for the occurrence and development of LEAD (22, 23). It is of great significance to determine the risk of LEAD in T2DM patients.

Inflammation, nutritional imbalance, and coagulation dysfunction play crucial roles in the occurrence and progression of arterial diseases. Based on this, a series of composite indices integrating multiple biomarkers have gradually become research hotspots in cardiovascular disease risk assessment, owing to their advantages of easy accessibility, convenient monitoring, and ability to comprehensively reflect the pathological status. As a novel integrated inflammatory marker, pan-immune inflammation value (PIV) comprehensively reflects the overall intensity of immunoinflammatory response by integrating peripheral blood neutrophil, lymphocyte, monocyte, and platelet counts. Its association with vascular diseases has been documented in relevant studies (24, 25). Platelet-to-neutrophil ratio (PNR) can simultaneously reflect the platelet activation state and neutrophil-mediated inflammatory response, and its correlation with vascular diseases has been reported (26). Fibrinogen-to-albumin ratio (FAR) takes into account both coagulation function (represented by fibrinogen) and the nutritional-inflammatory status (represented by albumin), and its association with vascular diseases has been confirmed (27, 28). However, the relationship between these comprehensive indices and LEAD in patients with T2DM is unclear. This study adopted a cross-sectional design. By analyzing the clinical data and laboratory indicators of patients with T2DM, we aimed to clarify the expression differences of the PIV, PNR, and FAR between T2DM patients complicated with LEAD and those without, and to explore the correlation of these indices with the risk of LEAD. The findings are expected to provide novel biomarker references for the early screening, risk stratification, and clinical intervention of LEAD in T2DM patients.

## 2 Materials and methods

### 2.1 Study cohort

**Figure 1:**
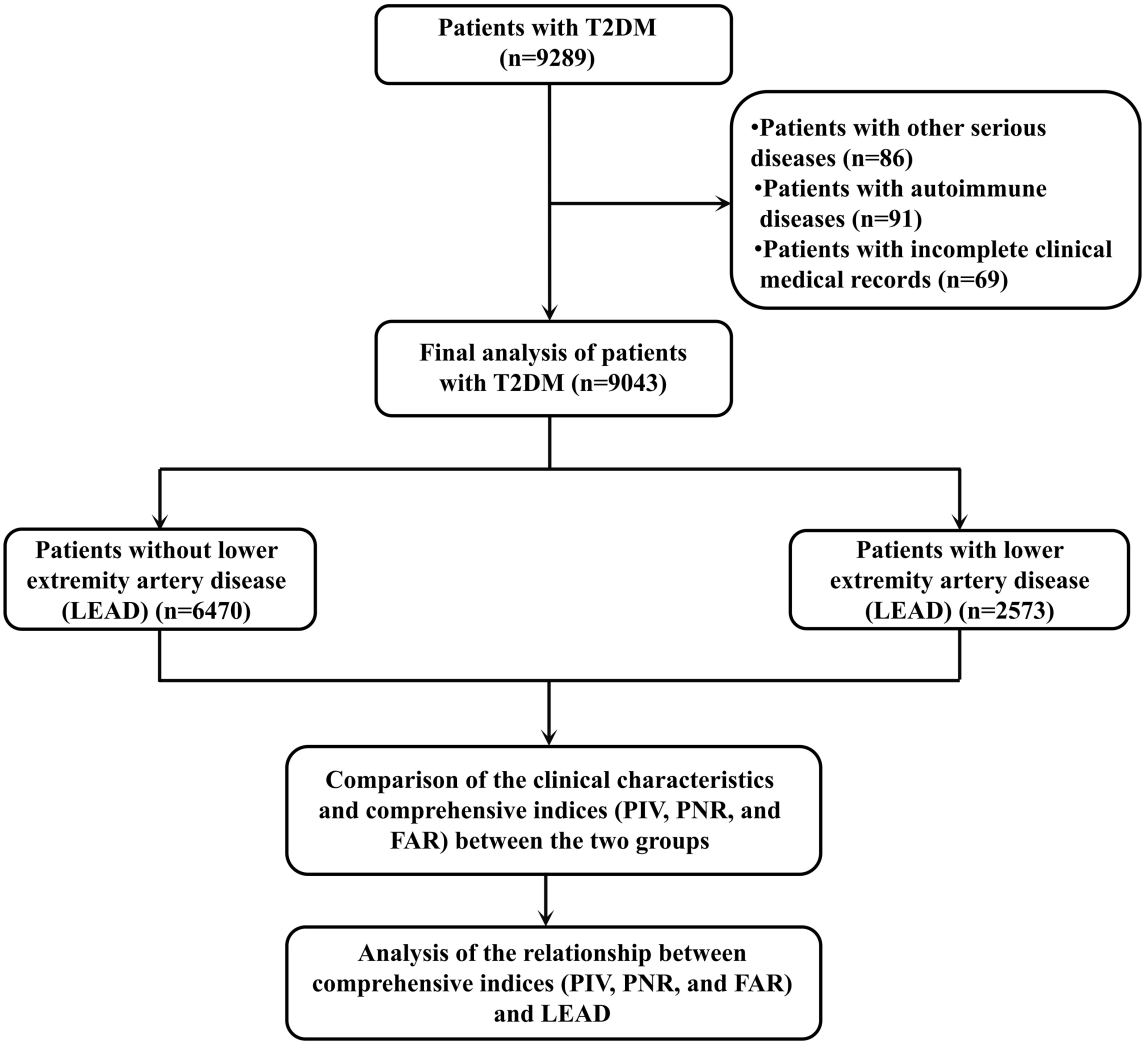
The flow chart of the present study.

This study conducted a retrospective analysis of patients with T2DM who visited Meizhou People’s Hospital from October 2020 to February 2025. This study was performed under the guidance of the Declaration of Helsinki and approved by the Ethics Committee of Medicine, Meizhou People’s Hospital. Patients who met the following conditions were included in this study: (1) age ≥18 years; (2) patients who meet the diagnostic criteria for T2DM set by the World Health Organization (WHO); and (3) patients with complete clinical medical information. Patients with the following conditions were excluded from this study: (1) patients with other serious diseases, severe organ failure, autoimmune diseases and other conditions; (2) patients with concurrent diseases that affect blood cell count, platelet, fibrinogen, and albumin levels; and (3) patients with incomplete clinical medical records. A total of 9043 patients were included in this study. The flowchart of this study is shown in **Figure 1**.

LEAD in patients with diabetes mellitus was defined as the presence of atherosclerotic stenosis or occlusion in the arteries of the lower extremities secondary to long-term hyperglycemia-induced vascular endothelial damage and metabolic disorders; the diagnostic criteria were based on the 2022 Guidelines for the Diagnosis and Treatment of Lower Extremity Arterial Disease in Diabetic Patients: (1) presence of typical clinical manifestations including intermittent claudication, rest pain, lower limb numbness, coolness or skin ulceration/gangrene; (2) ankle-brachial index (ABI) ≤ 0.90 or toe-brachial index (TBI) ≤ 0.70 at rest, or a decrease in ABI by ≥ 0.15 after exercise compared with baseline; (3) confirmation of vascular lesions via non-invasive vascular imaging techniques such as color Doppler ultrasound, computed tomography angiography (CTA) or magnetic resonance angiography (MRA), with evidence of ≥ 50% luminal stenosis in the lower extremity arteries or complete occlusion of the target vessel segment.

### 2.2 Data collection

The clinical data were collected, such as gender, age, body mass index (BMI), history of cigarette smoking, alcoholism, hypertension, cerebrovascular disease, diabetic peripheral neuropathy, and LEAD. Cerebrovascular diseases include cerebral hemorrhage, cerebral infarction, cerebral atherosclerosis, cerebral arteriovenous fistula, cerebral aneurysm, cerebral arteriovenous malformation, cerebral vascular stenosis, and cerebral vascular occlusion, and so on.

In line with Chinese criteria, BMI is divided into three grades: <18.5 kg/m^2^ (underweight), 18.5-23.9 kg/m^2^ (normal weight), and ≥24.0 kg/m^2^ (overweight) (29, 30). Patients with a history of hypertension are defined as those who have been clearly diagnosed with hypertension in the past or are currently undergoing oral antihypertensive drug treatment. Patients with a history of smoking refer to those who have smoked for at least one year or longer, with no less than one cigarette per day, or those who have been smoke-free for less than six months. Patients with a history of alcohol consumption refer to those who drink alcohol at least once a week.

### 2.3 Collection of laboratory test results and calculation of PIV, PNR, and FAR

The results of monocyte count, neutrophil count, platelet count, lymphocyte count, fibrinogen, and albumin were collected during the first hospital examination. The patient’s venous blood was collected before treatment, blood cell analysis was tested by Sysmex XE-2100 hematology analyzer (Sysmex Corporation, Japan). Plasma fibrinogen levels were measured via the Clauss method on a Sysmex CA-7000 automatic coagulation analyzer, while serum albumin concentrations were detected by the bromocresol green (BCG) method with a Roche Cobas c702 automatic biochemistry analyzer.

PIV, PNR, and FAR were calculated according to the following formula:


$$\begin{array}{l} \text{PIV}=\text{monocytecount}\times \text{neutrophilcount}\times \text{plateletcoun}t/\text{lymphocytecount}\\ \text{PNR}=\text{plateletcount}/\text{neutrophilcount}\\ \text{FAR}=\text{fibrinogen}/\text{albumin} \end{array}$$

### 2.4 Statistical analysis

Data analysis was performed using SPSS statistical software version 26.0 (IBM Inc., USA). Categorical variables are expressed as the number of cases (%), and compared between groups using the χ^2^ test. The optimal thresholds of PIV, PNR, and FAR for distinguishing patients with and without LEAD were determined by receiver operating characteristic (ROC) curve analysis. Logistic regression analysis was used to analysis the relationship of PIV, PNR, and FAR and LEAD adjusting for other covariates. *p* < 0.05.

## 3 Results

### 3.1 The clinical features of patients with T2DM

There were 5401 (59.7%) male and 3642 (40.3%) female patients; 1818 (20.1%) patients aged <60 and 7225 (79.9%) patients aged ≥60. There were 410 (4.5%) patients with underweight and 4322 (47.8%) with overweight. There were 931 (10.3%), 339 (3.7%), 4959 (54.8%), 1686 (18.6%), 931 (10.3%), and 1997 (22.1%) patients with cigarette smoking, alcoholism, hypertension, history of cerebrovascular disease, diabetic peripheral neuropathy, and LEAD, respectively. The median of PIV, PNR, and FAR was 336.84 (180.35, 765.83), 45.11 (30.44, 61.82), and 0.10 (0.08, 0.14), respectively (**Table 1**).

**Table 1:** The clinical features of the patients with type 2 diabetes mellitus (T2DM).

Clinical characteristics	Patients with T2DM (n=9043)
Gender	
Male, n(%)	5401 (59.7%)
Female, n(%)	3642 (40.3%)
Age (years)	
<60, n(%)	1818 (20.1%)
≥60, n(%)	7225 (79.9%)
BMI (kg/m^2^)	
Underweight, n (%)	410 (4.5%)
Normal weight, n (%)	4311 (47.7%)
Overweight, n (%)	4322 (47.8%)
Cigarette smoking	
No, n(%)	8112 (89.7%)
Yes, n(%)	931 (10.3%)
Alcoholism	
No, n(%)	8704 (96.3%)
Yes, n(%)	339 (3.7%)
Hypertension	
No, n(%)	4084 (45.2%)
Yes, n(%)	4959 (54.8%)
History of cerebrovascular disease	
No, n (%)	7357 (81.4%)
Yes, n (%)	1686 (18.6%)
Diabetic peripheral neuropathy	
No, n (%)	8112 (89.7%)
Yes, n (%)	931 (10.3%)
LEAD	
No, n (%)	7046 (77.9%)
Yes, n (%)	1997 (22.1%)
PIV, median (IQR)	336.84 (180.35, 765.83)
PNR, median (IQR)	45.11 (30.44, 61.82)
FAR, median (IQR)	0.10 (0.08, 0.14)

T2DM, type 2 diabetes mellitus; BMI, body mass index; LEAD, lower extremity artery disease; PIV, pan-immune inflammation value; PNR, platelet-to-neutrophil ratio; FAR, fibrinogen-to-albumin ratio; IQR, interquartile range. BMI is divided into three grades: <18.5 kg/m^2^ (underweight), 18.5-23.9 kg/m^2^ (normal weight), and ≥24.0 kg/m^2^ (overweight).

### 3.2 Comparison of the clinical features between patients with and without LEAD among T2DM patients

**Figure 2:**
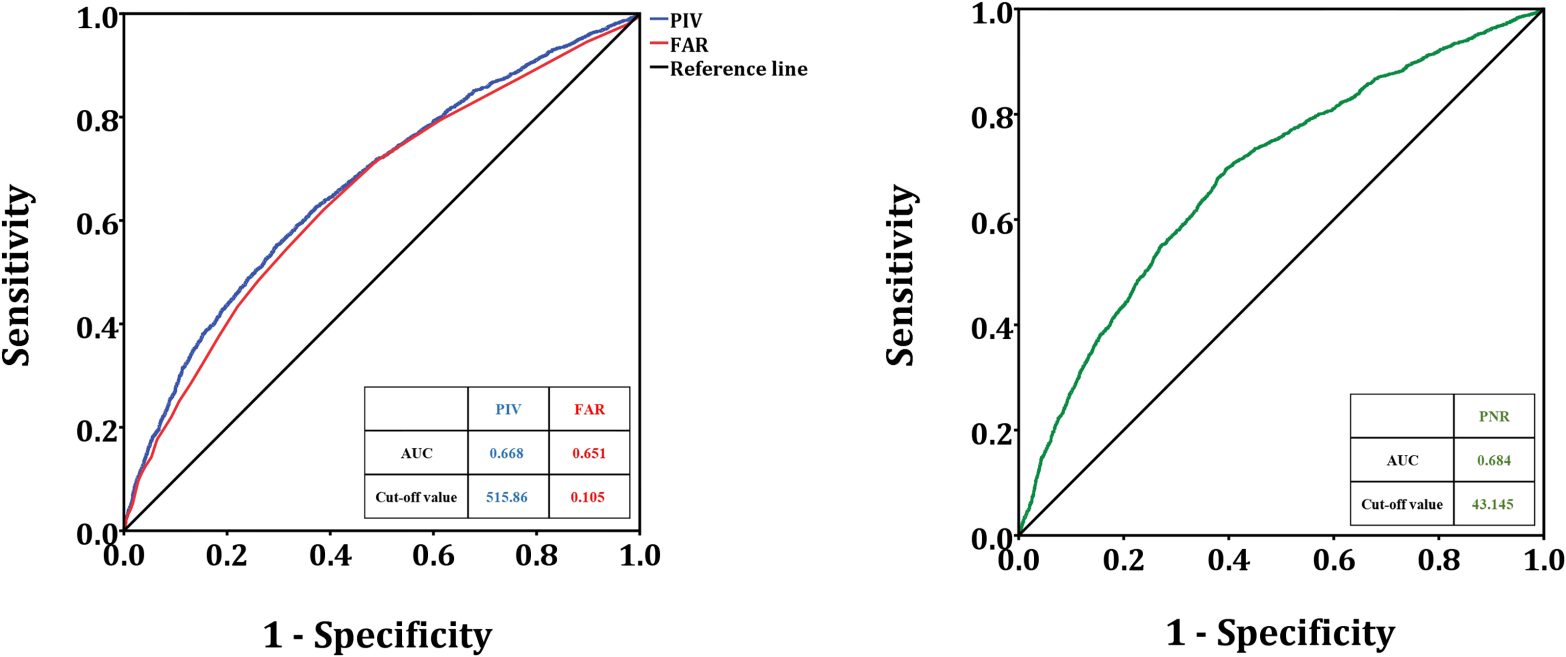
The ROC curve analysis of PIV, PNR, FAR to distinguish LEAD. PIV, pan-immune inflammation value; PNR, platelet-to-neutrophil ratio; FAR, fibrinogen-to-albumin ratio; LEAD, lower extremity artery disease.

The critical value of PIV was 515.86 (sensitivity 55.3%, specificity 70.3%, area under the ROC curve (AUC): 0.622), the PNR cutoff value was 43.145 (sensitivity 69.6%, specificity 60.5%, AUC: 0.642), the FAR cutoff value was 0.105 (sensitivity 62.1%, specificity 61.3%, AUC: 0.632) for distinguishing patients with and without LEAD were determined by ROC curve analysis (**Figure 2**).

In this study, there were 2573 (28.5%) T2DM patients with LEAD and 6470 (71.5%) patients without. There were statistically significant differences in the proportions of cigarette smoking (χ^2^ = 77.927, *p* < 0.001), alcoholism (χ^2^ = 12.266, *p* = 0.001), hypertension (χ^2^ = 173.214, *p* < 0.001), history of cerebrovascular disease (χ^2^ = 526.108, *p* < 0.001), diabetic peripheral neuropathy (χ^2^ = 1344.494, *p* < 0.001), high PIV (χ^2^ = 265.717, *p* < 0.001), low PNR (χ^2^ = 398.680, *p* < 0.001), and high FAR (χ^2^ = 313.501, *p* < 0.001) between T2DM patients with and without LEAD (**Table 2**).

**Table 2:** Comparison of the clinical features between patients with and without LEAD in patients with T2DM.

Clinical characteristics	Patients without LEAD (n=6470)	Patients with LEAD (n=2573)	*p* (χ^2^)
Gender			
Male, n(%)	3828 (59.2%)	1573 (61.1%)	0.087 (χ^2^ = 2.969)
Female, n(%)	2642 (40.8%)	1000 (38.9%)	
Age (years)			
<60, n(%)	1314 (20.3%)	504 (19.6%)	0.450 (χ^2^ = 0.596)
≥60, n(%)	5156 (79.7%)	2069 (80.4%)	
BMI (kg/m^2^)			
Underweight, n (%)	284 (4.4%)	126 (4.9%)	0.133 (χ^2^ = 4.036)
Normal weight, n (%)	3125 (48.3%)	1186 (46.1%)	
Overweight, n (%)	3061 (47.3%)	1261 (49.0%)	
Cigarette smoking			
No, n(%)	5919 (91.5%)	2193 (85.2%)	<0.001 (χ^2^ = 77.927)
Yes, n(%)	551 (8.5%)	380 (14.8%)	
Alcoholism			
No, n(%)	6256 (96.7%)	2448 (95.1%)	0.001 (χ^2^ = 12.266)
Yes, n(%)	214 (3.3%)	125 (4.9%)	
Hypertension			
No, n(%)	3203 (49.5%)	881 (34.2%)	<0.001 (χ^2^ = 173.214)
Yes, n(%)	3267 (50.5%)	1692 (65.8%)	
History of cerebrovascular disease			
No, n (%)	5647 (87.3%)	1710 (66.5%)	<0.001 (χ^2^ = 526.108)
Yes, n (%)	823 (12.7%)	863 (33.5%)	
Diabetic peripheral neuropathy			
No, n (%)	6282 (97.1%)	1830 (71.1%)	<0.001 (χ^2^ = 1344.494)
Yes, n (%)	188 (2.9%)	743 (28.9%)	
PIV			
<515.86	4517 (69.8%)	1329 (51.7%)	<0.001 (χ^2^ = 265.717)
≥515.86	1953 (30.2%)	1244 (48.3%)	
PNR			
<43.145	2560 (39.6%)	1615 (62.8%)	<0.001 (χ^2^ = 398.680)
≥43.145	3910 (60.4%)	958 (37.2%)	
FAR			
<0.105	4008 (61.9%)	1067 (41.5%)	<0.001 (χ^2^ = 313.501)
≥0.105	2462 (38.1%)	1506 (58.5%)	

T2DM, type 2 diabetes mellitus; BMI, body mass index; LEAD, lower extremity artery disease; PIV, pan-immune inflammation value; PNR, platelet-to-neutrophil ratio; FAR, fibrinogen-to-albumin ratio. BMI is divided into three grades: <18.5 kg/m^2^ (underweight), 18.5-23.9 kg/m^2^ (normal weight), and ≥24.0 kg/m^2^ (overweight).

### 3.3 Logistic regression analysis of risk factors associated with LEAD among T2DM patients

Univariate analysis showed that cigarette smoking (odds ratio (OR): 1.861, 95% confidence interval (CI): 1.619-2.140, *p* < 0.001), alcoholism (OR: 1.493, 95% CI: 1.191-1.870, *p* < 0.001), hypertension (OR: 1.883, 95% CI: 1.712-2.070, *p* < 0.001), history of cerebrovascular disease (OR: 3.463, 95% CI: 3.103-3.865, *p* < 0.001), and diabetic peripheral neuropathy (OR: 13.567, 95% CI: 11.466-16.053, *p* < 0.001), high PIV (OR: 2.165, 95% CI: 1.971-2.378, *p* < 0.001), low PNR (OR: 2.575, 95% CI: 2.343-2.829, *p* < 0.001), and high FAR (OR: 2.298, 95% CI: 2.093-2.522, *p* < 0.001) were significantly associated with LEAD in T2DM patients (**Table 3**).

**Table 3:** Univariate logistic regression analysis of risk factors associated with LEAD in T2DM.

Variables	OR	95% CI	*p* values
Gender (male vs. female)	1.086	0.989-1.192	0.085
Age (≥60 vs. <60, years)	1.046	0.933-1.173	0.440
BMI (kg/m^2^)			
Normal weight	1.000 (reference)	–	–
Underweight	1.169	0.938-1.457	0.165
Overweight	1.085	0.988-1.192	0.086
Cigarette smoking (yes vs. no)	1.861	1.619-2.140	<0.001
Alcoholism (yes vs. no)	1.493	1.191-1.870	<0.001
Hypertension (yes vs. no)	1.883	1.712-2.070	<0.001
History of cerebrovascular disease (yes vs. no)	3.463	3.103-3.865	<0.001
Diabetic peripheral neuropathy (yes vs. no)	13.567	11.466-16.053	<0.001
PIV (≥515.86 vs. <515.86)	2.165	1.971-2.378	<0.001
PNR (<43.145 vs. ≥43.145)	2.575	2.343-2.829	<0.001
FAR (≥0.105 vs. <0.105)	2.298	2.093-2.522	<0.001

T2DM, type 2 diabetes mellitus; BMI, body mass index; LEAD, lower extremity artery disease; PIV, pan-immune inflammation value; PNR, platelet-to-neutrophil ratio; FAR, fibrinogen-to-albumin ratio; OR, odds ratio; CI, confidence interval. BMI is divided into three grades: <18.5 kg/m^2^ (underweight), 18.5-23.9 kg/m^2^ (normal weight), and ≥24.0 kg/m^2^ (overweight).

Multivariate logistic regression analysis showed that cigarette smoking (OR: 1.802, 95% CI: 1.506-2.156, *p* < 0.001), hypertension (OR: 1.633, 95% CI: 1.460-1.826, *p* < 0.001), history of cerebrovascular disease (OR: 3.034, 95% CI: 2.678-3.437, *p* < 0.001), and diabetic peripheral neuropathy (OR: 18.983, 95% CI: 15.819-22.780, *p* < 0.001), high PIV (OR: 1.338, 95% CI: 1.181-1.515, *p* < 0.001), low PNR (OR: 2.234, 95% CI: 1.985-2.515, *p* < 0.001), and high FAR (OR: 1.676, 95% CI: 1.493-1.881, *p* < 0.001) were significantly associated with LEAD in T2DM patients (**Table 4**).

**Table 4:** Multivariate logistic regression analysis of risk factors associated with LEAD in T2DM.

Variables	OR	95% CI	*p* values
Cigarette smoking (yes vs. no)	1.802	1.506-2.156	<0.001
Alcoholism (yes vs. no)	0.788	0.584-1.064	0.120
Hypertension (yes vs. no)	1.633	1.460-1.826	<0.001
History of cerebrovascular disease (yes vs. no)	3.034	2.678-3.437	<0.001
Diabetic peripheral neuropathy (yes vs. no)	18.983	15.819-22.780	<0.001
PIV (≥515.86 vs. <515.86)	1.338	1.181-1.515	<0.001
PNR (<43.145 vs. ≥43.145)	2.234	1.985-2.515	<0.001
FAR (≥0.105 vs. <0.105)	1.676	1.493-1.881	<0.001

T2DM, type 2 diabetes mellitus; BMI, body mass index; LEAD, lower extremity artery disease; PIV, pan-immune inflammation value; PNR, platelet-to-neutrophil ratio; FAR, fibrinogen-to-albumin ratio; OR, odds ratio; CI, confidence interval. BMI is divided into three grades: <18.5 kg/m^2^ (underweight), 18.5-23.9 kg/m^2^ (normal weight), and ≥24.0 kg/m^2^ (overweight).

## 4 Discussion

In the early stage of LEAD, there are generally no obvious clinical symptoms. As arteriosclerosis progresses, ischemia and hypoxia of tissue cells cause a series of symptoms. Analyzing the risk factors of LEAD in patients with T2DM is helpful for the early detection and treatment of LEAD patients. In this study, overweight, cigarette smoking, hypertension, history of cerebrovascular disease, diabetic peripheral neuropathy, high PIV, low PNR, and high FAR were significantly associated with LEAD in T2DM patients.

PIV is an indicator comprehensively reflecting the immune inflammatory status of the body, showed a significant correlation with the occurrence and development of LEAD in patients with T2DM in this study. The long-term hyperglycemic environment in patients with T2DM can induce chronic and low-grade inflammatory responses, promoting the activation of immune cells and the release of a large amount of inflammatory factors (31). PIV integrates multiple parameters related to immune cells and inflammatory mediators. Its increase indicates that the body is in an excessive inflammatory state (32). Excessive inflammatory responses can damage vascular endothelial cells, leading to vascular endothelial dysfunction and undermining the integrity of the vascular wall (33). Meanwhile, inflammatory factors can also promote the migration of monocytes, and macrophages to the subintima of blood vessels, accelerate the formation of atherosclerotic plaques, and thereby increase the risk of LEAD (34). So, PIV may indirectly reflect the continuous damage of inflammation to the vascular wall of lower extremity arteries by reflecting the intensity of systemic inflammation, providing a basis for predicting the progression of LEAD.

PNR reflects the balance relationship between platelets and neutrophils and is also closely related to LEAD in patients with T2DM. Platelets are prone to activation in hyperglycemic environment (35). Activated platelets not only release substances such as thromboxane A_2_ (TXA_2_) and adenosine diphosphate (ADP) to promote platelet aggregation and form microthrombi, but also secrete inflammatory mediators to further intensify the inflammatory response (36). Neutrophils, as an important component of innate immunity, their increase indicates an enhanced inflammatory response in the body (37). A decrease in PNR indicates a reduction in the ratio of platelets to neutrophils, which may imply that the inflammatory response is dominant. Excessive inflammation accelerating the process of LEAD. In addition, the interactions between platelets and neutrophils, such as the formation of neutrophil extracellular traps (NETs), may also be involved in the injury and thrombosis process of lower extremity arterial vessels (38).

FAR integrates coagulation function and nutritional metabolic status and is of great significance in the occurrence and development of LEAD in patients with T2DM. Fibrinogen is a key component of the coagulation system. Hyperglycemic conditions can stimulate an increase in the liver’s synthesis of fibrinogen (39). An elevated concentration of fibrinogen in the blood will lead to an increase in blood viscosity and a slowdown in blood flow velocity, which is conducive to thrombosis (40). During the course of lower extremity vascular lesions, fibrinogen can interact with platelets at the site of vascular injury and participate in the formation of thrombosis. Meanwhile, fibrinogen can also regulate cell adhesion, migration and proliferation by binding to cell surface receptors, and affect the repair and remodeling process of the vascular wall. In patients with T2DM, due to metabolic disorders, kidney damage and other reasons, hypoalbuminemia often occurs, which can lead to dysfunction of vascular endothelial cells, affect the stability and repair ability of blood vessels, and at the same time weaken the body’s antioxidant and anti-inflammatory capabilities, aggravate oxidative stress and inflammatory responses, and thereby promote the occurrence and development of lower extremity vascular diseases (41). This state will accelerate the progression of atherosclerotic plaques in the lower extremities, increase the risk of plaque rupture and thrombosis, and thereby promote the occurrence and deterioration of LEAD. Celebi et al. found that FAR was higher in patients with LEAD than those without LEAD (42). Liu et al. found that neutrophil to lymphocyte ratio (NLR) and platelet to lymphocyte ratio (PLR) were associated with LEAD in T2DM (43).

As for the relationship between traditional risk factors and LEAD, there have been some studies. A few studies unanimously hold that smoking and hypertension are risk factors for the development of artery disease (44–46). Age was associated with the risk of LEAD in patients with T2DM (47), however, similar results were not obtained in this study. Some studies suggested that male is a risk factor of the development of artery disease (44, 48). However, this study did not find a relationship between gender and LEAD. Obesity was associated with LEAD in patients with T2DM (49). In addition, Zhu et al. found that cerebrovascular diseases are risk factors for lower extremity atherosclerotic diseases in patients with T2DM (50). In this study, history of cerebrovascular disease was associated with LEAD in patients with T2DM.

In this study, we found that overweight, cigarette smoking, hypertension, history of cerebrovascular disease, diabetic peripheral neuropathy, high PIV, low PNR, and high FAR were significantly associated with LEAD in T2DM patients. However, this study still has limitations. Firstly, the study was a cross-sectional one, making it difficult to clarify the causal relationship and dynamic change patterns between PIV, PNR, FAR and LEAD. Secondly, the synergistic mechanism between these indicators and other risk factors (such as dyslipidemia, elevated blood pressure, etc.) has not been deeply explored. Thirdly, there is a lack of clinical prognosis observation after intervention for these indicators. Finally, the study population was recruited from a relatively homogeneous source, which may have introduced selection bias. Therefore, the extrapolability of these findings needs to be further verified in cohorts of patients with T2DM from diverse geographical regions and ethnic backgrounds. Future studies can conduct prospective cohort studies to dynamically monitor the changes of these indicators in patients with T2DM and their relationship with the occurrence and development of LEAD. Explore its interaction with other risk factors by using multi-omics techniques; And clinical studies based on indicator intervention were carried out to evaluate the feasibility of improving the prognosis of LEAD in patients with T2DM by regulating PIV, PNR and FAR, providing more precise strategies for disease prevention and treatment.

## 5 Conclusion

Cigarette smoking, hypertension, history of cerebrovascular disease, diabetic peripheral neuropathy, high PIV, low PNR, and high FAR were significantly associated with LEAD in T2DM patients. In particular, PIV, PNR, and FAR can serve as potential inflammation- and coagulation-related biomarkers for assessing the risk of LEAD in T2DM patients, thereby providing a reference basis for clinical early screening, risk stratification, and intervention. As a conveniently accessible set of indicators, this study provides a convenient method for clinicians to evaluate the occurrence of LEAD in patients with T2DM.

## Data Availability

The original contributions presented in the study are included in the article/supplementary material. Further inquiries can be directed to the corresponding author.
